# Management of Auricular Hematoma With Bolster Versus Quilting: A Retrospective Cohort Study

**DOI:** 10.1002/lary.70680

**Published:** 2026-06-11

**Authors:** Christie F. Cheng, Andrew Neevel, Michael J. Brenner, Shannon F. Rudy

**Affiliations:** ^1^ Department of Otolaryngology—Head and Neck Surgery University of Michigan USA

**Keywords:** auricular hematoma, bolster, mattress suture quilting, practice patterns, pressure dressing

## Abstract

**Objective:**

Auricular hematomas commonly result from soft tissue trauma and require prompt drainage and pressure dressing to prevent recurrence and deformity. Both bolster and quilting suture techniques are used post‐drainage, but direct comparisons are lacking. The objective of this study is to compare outcomes between bolster dressings and through‐and‐through mattress quilting sutures in the management of auricular hematomas.

**Methods:**

A 15‐year retrospective chart review was conducted for patients age > 10 years presenting to the Emergency Department with auricular hematoma. Exclusion criteria included developmental delay, anticoagulant use, infectious etiology, or subsequent diagnosis of relapsing polychondritis. Cases were analyzed for complications and recurrence.

**Results:**

Forty‐three patients met inclusion criteria (95% male; mean age 33 years). Thirty (73%) were managed with bolsters and 13 (27%) with quilting sutures. Recurrent hematoma occurred in 30% (9/30) of the bolster group and 0% (0/13) of the quilting group, including 11 primary cases and 2 salvage cases (*p* = 0.036). No significant differences were observed between the two groups in terms of complications (*p* = 1.0) or scarring (*p* = 0.29).

**Conclusions:**

Quilting suture technique had lower rates of recurrence compared to bolster dressings for auricular hematoma management. These findings support quilting as an acceptable option following hematoma drainage, with controlled studies necessary to determine comparative efficacy.

**Level of Evidence:**

3.

## Graphical abstract

This retrospective cohort study compared outcomes between bolster dressings and through‐and‐through mattress quilting sutures in the management of auricular hematoma in 43 patients. Recurrent hematoma occurred in 30% of the bolster group and 0% of the quilting group, with no significant differences in complications observed between the two groups. These findings support quilting as an acceptable option following hematoma drainage, with controlled studies necessary to determine comparative efficacy.

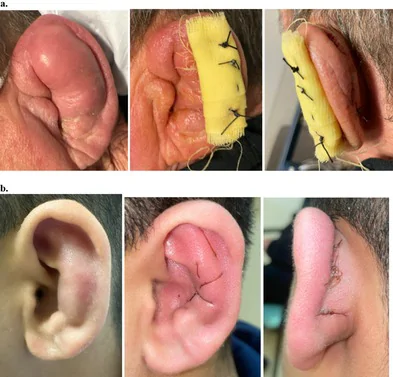

## 1 Introduction

Auricular hematomas classically result from blunt trauma to the external ear but can also occur spontaneously, often in the setting of anticoagulation, infection, or autoimmune disease. Timely hematoma evacuation and prevention of blood re‐accumulation are necessary to prevent infection and late complications such as cauliflower ear. The pathogenesis of this deformity involves accumulation of blood between the perichondrium and cartilage, which deprives the cartilage of nutrients and results in chondronecrosis and fibrocartilaginous deposition [1, 2].

The techniques for treating auricular hematomas aim to obliterate the potential space and reduce the risk for re‐accumulation. Bolsters may involve dental rolls, gauze, molded splints, buttons, or magnets [3, 4, 5, 6]. Additional techniques include placement of drains [1, 7], fibrin glue [8], cartilage fenestration [9, 10], and through‐and‐through mattress sutures [11, 12, 13].

Mattress suture quilting is often faster and simpler than bolster placement with an overall good outcome [13, 14]. The technique involves suturing through‐and‐through the auricle to tack the anterior and posterior skin and soft tissue to the auricular cartilage. Securing bolsters is more time‐consuming and cumbersome to patients but may distribute pressure more evenly [1, 2]. This study analyzed comparative outcomes with bolster versus quilting techniques.

## 2 Materials and Methods

### 2.1 Study Design and Patient Selection

This study was designated as exempt by the Institutional Review Board at the University of Michigan (HUM00270793). A 15‐year retrospective chart review was performed identifying patients with auricular hematoma who presented to the Emergency Department and were managed by Otolaryngology. The electronic health record was queried using DataDirect, an institution‐specific research database, and patients were identified using ICD‐9 and ICD‐10 diagnosis codes for auricular hematoma (380.0 and H61.1). Patients were excluded if they were under the age of 10, had a diagnosis of developmental delay, were on anticoagulation, presented with infection/abscess, or had a diagnosis of relapsing polychondritis at time of presentation or subsequently thereafter.

All procedures included were performed by an Otolaryngology resident in the Emergency Department or directly overseen or performed by Otolaryngology faculty in clinic. All residents at this institution completed at least 2 separate simulations for auricular hematoma management in their intern year, which included high‐fidelity practice with the bolster technique.

### 2.2 Chart Review and Analysis

Chart review was performed and data on demographics, hematoma location, injury mechanism, management, antibiotics, follow‐up, and outcomes were collected and analyzed. A bolster dressing was defined as the application of a Vaseline or xeroform gauze or cotton‐based dressing secured with suture to apply even pressure to the area following hematoma drainage. Mattress suture quilting was defined as through‐and‐through suturing of the skin and cartilage in an anterior–posterior manner to obliterate the potential space after hematoma drainage (Figure 1). Recurrence was defined as clinically evident re‐accumulation of auricular hematoma on physical examination at any follow‐up, with or without need for repeat intervention. Fisher's exact test was used for statistical analysis with a *p* < 0.05 considered to be statistically significant. In instances of salvage procedures (e.g., placement of quilting sutures to manage blood re‐accumulation after bolster removal), the data were recorded and analyzed for each technique used, respectively. For all other analyses, attribution was based on initial treatment technique.

**FIGURE 1:**
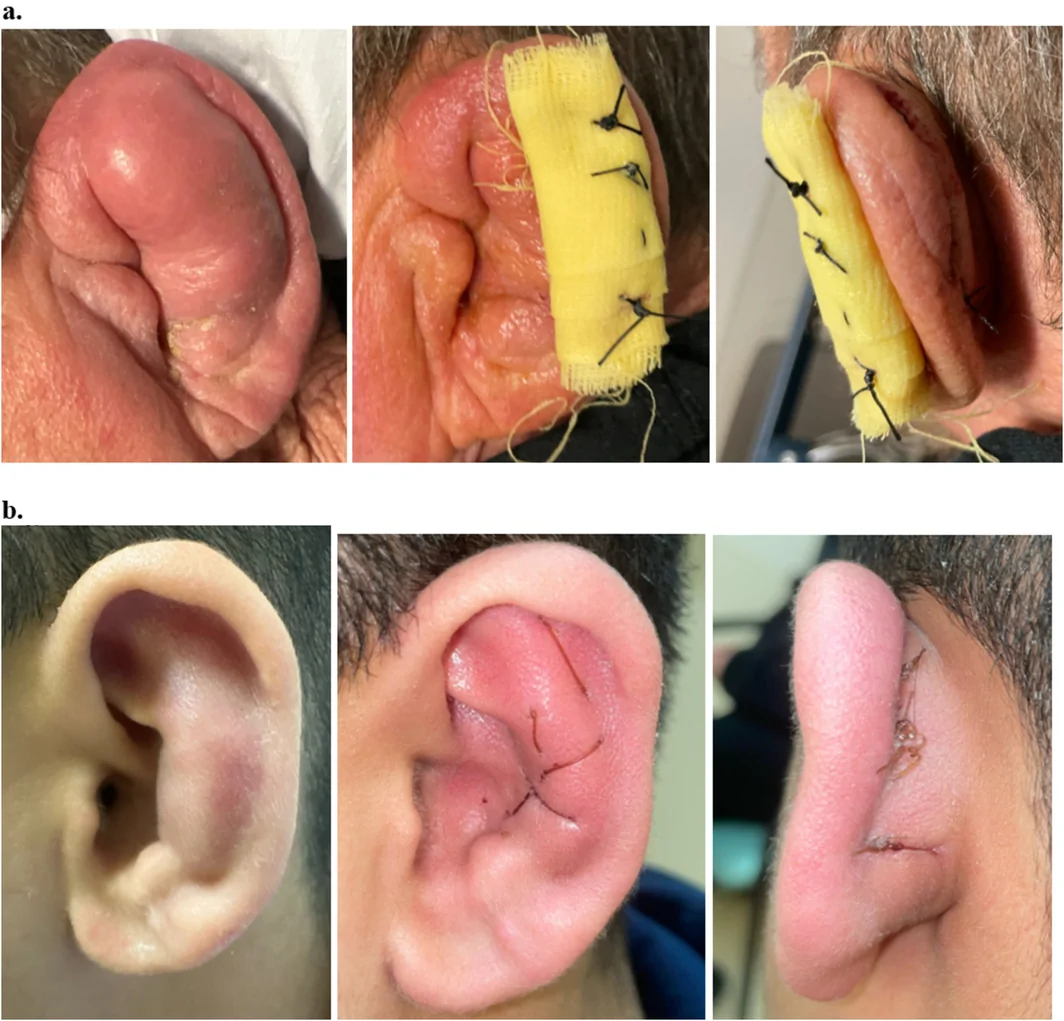
(a) Bolster example. (b) Mattress suture quilting example.

## 3 Results

We identified 43 patients who presented to our institution's Emergency Department with auricular hematoma who were managed by Otolaryngology. Mean age was 33 years (range 11–89 years), and 91% of cases (*n* = 39) occurred in male patients. Twenty‐four cases involved the left ear, and 19 involved the right ear. No patients presented with bilateral auricular hematomas. In 2 cases, blood re‐accumulation following bolster removal was salvaged with mattress suture quilting. The most common injury mechanism was an athletic‐related trauma (53%), followed by accidents, such as falls or motor vehicle collisions (21%), and general trauma, such as assault or picking (14%). Forty patients were treated with antibiotics, with the most common being fluoroquinolones (*n* = 25) followed by beta‐lactam antibiotics (*n* = 13) (Table 1).

**TABLE 1:** Patient demographics and baseline characteristics.

Mean age (years)		33 (range 11–89)
Sex	Male	39
	Female	4
Race/ethnicity	White	32
	Black	3
	Latino	2
	Asian	5
	Middle eastern	1
Laterality	Left	24
	Right	19
	Bilateral	0
Location (relative to superior external auditory canal)	Superior	20
	Inferior	17
	Both	6

Average time to presentation (days)		4 (range 0–21)
Type of injury	Athletics (e.g., wrestling, martial arts, sports)	23
	Trauma (e.g., assault, rubbing/picking, animal bite)	6
	Accident (e.g., fall, motor vehicle)	9
	Other (e.g., iatrogenic or unknown)	5
Antibiotics	Fluroquinolone	25
	Beta‐lactam	13
	Combination fluoroquinolone with beta‐lactam	1
	Bactrim	1
	No antibiotics	3
Time to follow‐up	Within 5 days	13
	5–10 days	23
	> 10 days	3
	None	4

Thirty patients (70%) were managed with bolster application. Among this cohort, the mean age was 31 years (range 11–89 years) and 93% were male. The hematoma spanned the entire auricle in 3 patients (10%), was superior to the external auditory canal (EAC) in 13 patients (43%), and inferior to the EAC in 14 patients (47%). Athletics‐related trauma accounted for 57% of cases. The average time to presentation was 4.4 days (range 0–21 days). The median time to follow‐up was 6 days (range 3–17 days) with 1 patient lost to follow‐up (Table 2).

**TABLE 2:** Bolster versus mattress suture quilting outcomes.

		Bolster (*n* = 30)	Quilted (*n* = 13)	*p*
Mean age (range)		31 (11–89)	38 (13–81)	
Distance to hospital	Less than 2 h driving	23	10	
	2+ hr. of driving	3	0	
	Out of state	4	3	
Sex	Male	28	11	
	Female	2	2	
Type of injury	Athletics (e.g., martial arts, sports)	17	6	
	Trauma (e.g., assault, rubbing/picking, animal bite)	4	2	
	Accident (e.g., fall, motor vehicle)	5	4	
	Other	4	1	
Location (relative to superior external auditory canal)	Superior	13	9	
	Inferior	14	1	
	Both	3	3	
Time to presentation (mean, range)		4.4 days (0–21)	2.5 days (0–12)	
Time to follow‐up (median, range)		6 days (3–17)	6 days (2–12)	
Socioeconomic concerns	Yes	0	4	
Recurrence	Yes	9	0	*p* = 0.036*
	No	20	10	
	Unknown (no f/u)	1	3	
Need for further procedures	Yes	8	0	*p* = 0.089
	No/Patient declined	21	10	
	Unknown (no f/u)	1	3	
Compilations	Yes	2	0	*p* = 1.0
	No	27	10	
	Unknown (no f/u)	1	3	
Scarring/deformity (initial visit)	Yes	4	0	*p* = 0.29
	No	25	10	
	Unknown (no f/u)	1	3	

* *p* < 0.05 was considered statistically significant.

Eleven patients (26%) were initially managed with mattress suture quilting and 2 patients initially treated with bolster received salvage with mattress suture quilting. Among this cohort, the mean age was 38 years (range 13–81 years) and 85% were male. The hematoma spanned the entire auricle in 3 patients (23%), was superior to the EAC in 9 patients (69%), and inferior to the EAC in 1 patient (8%). Similarly to the bolster cohort, athletics‐related trauma accounted for the majority of cases (46%). The average time to presentation was 2.5 days (range 0–12 days). The median time to follow‐up was 6 days (range 2–12 days) with 3 patients lost to follow‐up (Table 2).

Thirty percent (*n* = 9) of the bolster cohort developed recurrent hematomas, approximately half (*n* = 4) of which were multiply recurrent. One patient who developed recurrence had initially been treated with bolster and quilting sutures. Five patients underwent repeat incision and drainage (I&D) followed by bolstering, 1 patient declined additional procedures, 1 patient was treated with aspiration alone, and 2 patients were ultimately managed with I&D followed by quilting. This was compared to a 0% recurrence rate in the mattress suture quilting group (*p* = 0.036). One patient had also received an adjunct compression dressing; however, he was lost to follow‐up and excluded from recurrence analysis. There was no significant difference between the two techniques in terms of complications at time of follow‐up, such as residual granulating wounds (*p* = 1.0) or scarring (*p* = 0.29), and there were no patients that developed infection after intervention. There was no definitive documentation of cauliflower ear in any of the cases and no referral to Otolaryngology for management of such (Table 2).

## 4 Discussion

Although bolstering is generally regarded as the gold standard for managing auricular hematoma, our 15‐year experience supports mattress suture quilting as an effective option for hematoma resolution in selected low‐risk patients. The rationale for selecting bolster versus quilting technique was not consistently documented and likely reflected clinician preference as well as clinical factors. While we excluded patients in whom treatment selection may have been influenced by specific factors (e.g., anticoagulation, developmental delay), residual selection bias remains an inherent limitation of this retrospective design. Mattress suture quilting had a statistically significant lower recurrence rate compared to bolster application (0% compared to 30%, respectively, *p* = 0.036), and there was no significant difference in complications or scarring at initial follow‐up. Long‐term outcomes, such as the development of cauliflower ear, were not systematically captured in this cohort and highlight the need for prospective studies with standardized long‐term follow‐up.

The comparability of patient populations and presenting disease characteristics between groups is an important consideration. Although the cohort size was insufficient to permit propensity matching, both groups were predominantly male patients under the age of 40 who presented within a few days of injury. In both cohorts, most auricular hematomas were limited to either the superior or inferior aspect of the auricle, although a subset in each group involved the entire auricle (10% in the bolster group and 23% in the quilting group). The mean time to presentation was 2.5 days (range 0–12 days) in the quilting group compared with 4.4 days (range 0–21 days) in the bolster group. Earlier intervention in the quilting group may have contributed to more favorable tissue conditions for hematoma resolution and potentially lower recurrence. However, given the retrospective design, this difference may also represent unmeasured selection bias, and causal inference cannot be established.

Our findings support existing literature concluding that mattress sutures are effective in preventing hematoma recurrence. Giles and colleagues performed a retrospective study in 2007 comparing mattress sutures, aspiration alone, and I&D followed by drain placement in preventing recurrence. The authors identified 22 patients with auricular hematoma and found a 5.3% recurrence rate in patients managed with sutures compared to a 75% recurrence for needle drainage alone and 20% recurrence for I&D followed by drain placement [11]. Mattress suture quilting may be helpful in the setting of concave anatomy or the need for a low‐profile compressive technique.

A limitation of prior literature assessing quilting sutures is a lack of comparator. Kakarala and colleagues evaluated the efficacy of mattress sutures to manage auricular hematoma recurrence after bolster placement [12]. They identified 28 patients with recurrent auricular hematomas who underwent I&D followed by mattress suture quilting. None of these patients developed recurrence, and cosmesis was judged as excellent by both the patient and surgeon [12]. Roy and Smith treated 8 professional fighters with auricular hematoma with I&D followed by mattress suture quilting. They did not apply bolsters and found that 100% of hematomas resolved without infection, complication, or need for further intervention [13]. Shakeel and colleagues similarly treated 15 patients with auricular hematoma, 10 of which were recurrent, with I&D followed by mattress suture quilting. They also did not utilize bolsters or external compression and found good resolution without significant recurrence [14].

A recent systematic review by Saad and colleagues supports I&D followed by pressure dressing application to prevent recurrence and maintain ear contour, specifically with mattress sutures yielding the best contour restoration among drainage techniques [15]. They also recommend early follow‐up within 24–48 h to monitor for re‐accumulation and longer term follow‐up within 2–4 weeks to assess cosmetic outcome [15]. In our study, no patients had documentation of cauliflower ear and none were referred to Otolaryngology for management of such. However, the limitations of this retrospective study design highlight the need for prospective studies assessing long‐term outcomes.

Our study is, to our knowledge, the first direct comparison between bolster and mattress suture quilting in auricular hematoma outcomes. While our sample sizes are small, the finding that mattress suture quilting had fewer recurrences compared to bolstering is significant because of several challenges with bolster dressings. Bolster dressings are meant to fix the soft tissue in place and provide even, consistent compression; however, the application may be challenging due to multi‐subsite involvement, slippery bolstering materials, or patient tolerance. Bolstering may obscure visual feedback confirming adequate pressure which could result in higher recurrence, especially with less experienced Otolaryngology residents. There is also greater technical challenge with bolsters, including the risk of needlestick injury when driving suture through cotton or gauze dressings. In this cohort, all residents had been trained with high‐fidelity simulations at least twice, which we would expect to reduce user error related to limited experience; however, most residents may not have the benefit of such training simulations.

Mattress suture quilting typically requires fewer supplies and involves specifically placed, through‐and‐through stitches to re‐approximate the skin to the cartilage and obliterate the potential space. Mattress sutures could be considered in lieu of or in conjunction with bolstering for targeted pressure application for hematomas spanning multiple concave sites or for patients at increased risk for recurrence, for example those on anticoagulation. They could also be considered in patients for which a lower‐profile dressing is desirable, such as those with developmental delay or in young children, in which mattress sutures may obviate the need for a bolster and subsequent removal or allow for a smaller bolster to be applied. Absorbable suture may also facilitate easier monitoring of the surgical site and can make video‐visit follow‐up more feasible for patients who live far away compared to bolsters which necessitate an in‐person visit.

Limitations of this study include the small sample size, retrospective nature, and lack of randomization, though baseline characteristics between the bolster and quilting groups were similar. We were also unable to assess time, expense, or resource utilization between techniques. There is also a risk of bias in which quilting may have been performed for either more or less severe cases, patient preference, or other social factors, necessitating cautious interpretation of findings and the need for controlled studies. Future directions include a randomized prospective study to compare the two techniques, assess long‐term outcomes, and assess these techniques in young children and patients on anticoagulation.

## 5 Conclusion

In a retrospective cohort of patients with auricular hematoma, the recurrence rate was lower in patients managed with mattress suture quilting compared to bolster placement with similar cosmetic outcomes. Because quilting is less resource‐ and time‐intensive and less cumbersome to patients, it may be considered a reasonable alternative to bolster placement in select low‐risk patients. Future randomized, controlled studies are necessary to determine comparative efficacy.

## Funding

The authors have nothing to report.

## Conflicts of Interest

The authors declare no conflicts of interest.

## Data Availability

The data that support the findings of this study are available from the corresponding author upon reasonable request.
